# Nanoparticles of *Lactiplantibacillus plantarum* K8 Reduce *Staphylococcus aureus* Respiratory Infection and Tumor Necrosis Factor Alpha- and Interferon Gamma-Induced Lung Inflammation

**DOI:** 10.3390/nu15224728

**Published:** 2023-11-09

**Authors:** Jonghyo Hong, Minseong Son, Jaeeun Sin, Hangeun Kim, Dae-Kyun Chung

**Affiliations:** 1 Graduate School of Biotechnology, Kyung Hee University, Yongin 17104, Republic of Korea; liil12@naver.com (J.H.); minseong9071@gmail.com (M.S.); jayiel821@khu.ac.kr (J.S.); 2 Research and Development Center, Skin Biotechnology Center Co., Ltd., Yongin 17104, Republic of Korea

**Keywords:** *Lactiplantibacillus plantarum* K8, lysates, probiotics nanoparticles, lung infection, *Staphylococcus aureus*, lung inflammation

## Abstract

Multiple studies have confirmed that *Lactiplantibacillus plantarum* has beneficial effects in respiratory diseases, including respiratory tract infections, asthma, and chronic obstructive pulmonary disease. However, the role of *L. plantarum* lysates in respiratory diseases is unclear. *Staphylococcus aureus* infects the lungs of mice, recruits immune cells, and induces structural changes in alveoli. Lung diseases can be further aggravated by inflammatory cytokines such as CCL2 and interleukin (IL)-6. In in vivo studies, *L. plantarum* K8 nanoparticles (K8NPs) restored lung function and prevented lung damage caused by *S. aureus* infection. They inhibited the *S. aureus* infection and the infiltration of immune cells and prevented the increase in goblet cell numbers in the lungs of *S. aureus*–infected mice. K8NPs suppressed the expression of CCL2 and IL-6, which were increased by the combination treatment of tumor necrosis factor alpha and interferon gamma (TI), in a dose-dependent manner. In in vitro studies, the anti-inflammatory effect of K8NPs in TI-treated A549 cells and TI-injected mice occurred through the reduction in activated mitogen-activated protein kinases and nuclear factor kappa-B. These findings suggest that the efficacy of K8NPs in controlling respiratory inflammation and infection can be used to develop functional materials that can prevent or alleviate respiratory diseases.

## 1. Introduction

Respiratory diseases are pathological conditions that occur in the respiratory organs and tissues, making gas exchange difficult. These can range from mild illnesses such as colds, influenza, and pharyngitis to life-threatening illnesses such as bacterial pneumonia, pulmonary embolism, tuberculosis, acute asthma, lung cancer, and severe acute respiratory syndrome (SARS) [1]. Chronic exposure to indoor allergens such as dust mites, pets, bacteria, and mold has been linked to asthma development. The specific role of allergens in the pathogenesis of allergic respiratory diseases is being studied because advances in molecular biology and omics have enabled the identification, replication, and expression of new indoor allergens [2]. Additionally, air pollutants can cause respiratory diseases by promoting oxidative stress, inducing inflammatory reactions, and lowering immunity [3]. *Staphylococcus aureus* is a Gram-positive bacterium and allergen that causes respiratory diseases. It is commonly present in the intestines and skin, as well as in the nose and respiratory tract. It can exist as both a normal flora and a pathogen, and can cause skin infections, respiratory infections, sinusitis, and food poisoning under certain circumstances [4]. The ability of *S. aureus* to adapt to a variety of respiratory environments has accelerated its emergence as a respiratory pathogen [5,6]. *S. aureus* enterotoxins (SEs) act as superantigens in the pathogenesis of several chronic airway diseases, which can induce an intense T cell activation through the release of interleukins (ILs)—4, 5, and 13. SEs can promote a polyclonal IgE response and an eosinophilic inflammation [6]. Another factor contributing to a respiratory disease is the increase in inflammatory responses triggered by damaged and dead microbial pathogens or host cells [7]. Increased expression of interleukin (IL)-6, IL-1β, tumor necrosis factor alpha (TNF-α), and IL-8 was measured in sputum, and this expression further increased during exacerbation [8]. Recent studies suggest that IL-6 plays an active role in the pathogenesis of COPD and asthma, and IL-6 could be a direct target in treatments of these and other chronic lung diseases [9]. Monocyte chemoattractant protein-1 (MCP-1/CCL2) is essential for H7N9 virus infection, which can result in SARS and severe forms of acute lung injury that contribute to morbidity and mortality [10].

Although oral corticosteroids are commonly used to treat asthma, they can cause systemic adverse effects including immune suppression, osteoporosis, diabetes, ocular disorders, and respiratory infections [11]. As an alternative, attempts to develop plant-derived functional materials that are effective for various diseases, including respiratory ailments, are being carried out as a national project [12,13,14]. For similar reasons, research is being conducted to develop probiotic substances that are effective in treating respiratory diseases. For example, the administration of some *Lactobacillus* species can relieve symptoms of respiratory diseases such as airway infections, asthma, lung cancer, and cystic fibrosis in both animal models and clinical trials [15]. Post-biotics such as lipids, proteins, carbohydrates, and more complex compounds isolated from probiotics can regulate a host’s immune system and help to regulate its physiological functions such as immuno-inflammatory actions, anti-obesity resistance, cholesterol reduction, hypertension control, and anti-cancer and anti-oxidative responses [16]. *Lactiplantibacillus plantarum* K8 nanoparticles (K8NPs), a form of post-biotics, contain various immunomodulatory factors and contribute to the maintenance of host homeostasis. 

Oral administration of nanoparticles obtained by pulverizing *L. plantarum* K8 can alleviate sepsis caused by a lipopolisaccharide (LPS) and alleviate an atopic skin disease by maximizing their moisturizing mechanisms [17,18]. Cell lysates of *L. plantarum* attenuate the poly I:C-induced phosphorylation of extracellular signal-regulated kinase (ERK) and c-Jun-terminal kinase (JNK) and the activation of nuclear factor kappa-B (NF-κB), and this results in the inhibition of IL-8 production in the human intestinal epithelial cell line HT-29 [19]. In addition, probiotic lysates contribute to improvements in oral health by attenuating periodontal inflammation and dental caries [20] and inducing re-epithelialization, anti-inflammatory, and anti-pathogenic effects [21,22,23]. They also have anti-adipogenic effects, making them potentially useful drugs for the treatment of obesity [24]. However, few studies have been conducted on the use of probiotic lysates to alleviate respiratory diseases. Here, we identify the role of K8NPs in respiratory infections and inflammation through in vivo studies using *S. aureus* infection models and in vitro studies using inflammation-inducing cell lines.

## 2. Materials and Methods

### 2.1. Preparation of K8 Nanoparticles, K8NPs

*L. plantarum* K8 cells isolated from Kimchi (KCTC 10887BP) were cultured in 2 L of MRS broth (BD Bioscience, San Jose, CA, USA) at 37 °C overnight, after which the cells were harvested using centrifugation at 5000× *g* for 10 min. Cells were resuspended in sterilized water and disrupted five times at 27,000 psi with a microfluidizer. Disrupted *L. plantarum* K8 cells were freeze-dried and resuspended in phosphate-buffered saline (PBS) at a concentration of 10^11^ colony-forming units (CFU)/mL for subsequent experiments (1 × 10^11^ CFU of K8NPs corresponds to 333 mg of the reference raw material, LTA-K8, and to 0.37 mg of N-acetylglucosamine (GlcNAc), an indicator substance of probiotics lysates).

### 2.2. In Vivo Study

The mice were cared for and used in accordance with guidelines set by the animal ethics committee of ChemOn Inc. Specific approval for the mouse experiments was obtained for the protocol (CHEM-2022-IA0701-00, 9 August 2022) from the Institutional Animal Care and Use Committee at ChemOn Inc. (Suwon, Republic of Korea). Male BALB/c mice (5 weeks old, *n* = 6/group) were obtained from Narabiotech (Seoul, Republic of Korea). Mice were housed in individual cages at 24 ± 2 °C and 50 ± 10% moisture condition and fed nutritionally balanced rodent food (Central Lab. Animal Inc., Seoul, Republic of Korea) and sterilized water. All reasonable efforts were made to ameliorate suffering, including the use of anesthesia for painful procedures. 

#### 2.2.1. Bacterial Culture and Infection of Mice

*S. aureus* colonies, purchased from the American Type Culture Collection (ATCC25923), were amplified by incubating them in a BHI medium (HiMedia Laboratories, Maharashtra, India) at 37 °C. The next day, the bacteria were sub-cultured in a fresh BHI medium for an additional 3–4 h until they reached a 600 nm optical density of 0.5. Cultured bacteria were collected using centrifugation at 5000× *g* for 10 min. After washing twice with fresh sterile PBS, the *S. aureus* cells were resuspended in PBS. *S. aureus* nasal inhalation was conducted as described in a previous study with minor modifications [25]. Mice were infected intra-nasally with various concentrations of *S. aureus* (8 × 10^8^ to 3.2 × 10^7^ CFU/50 µ PBS/mouse). In other experiments, mice were first orally administered with K8NPs (1 × 10^6^ to 1 × 10^8^ CFU/mouse) for 4 weeks, followed by the inhalation of *S. aureus* (3.2 × 10^7^ CFU/50 μL PBS/mouse) or the intraperitoneal injection of TI (250 µg/kg). The survival rate of mice was monitored over 7 days at 12 h intervals. Mice sera were collected for analysis of CCL2 and IL-6 levels. Lung tissues were obtained and fixed with 4% formaldehyde for histological analysis. Bronchoalveolar lavage fluid (BALF) was collected and washed with PBS, and the contents of the BALF were spread on brain heart infusion (BHI) agar plates to count the CFUs of *S. aureus*.

#### 2.2.2. Enzyme-Linked Immunosorbent Assay (ELISA) Analysis Using Mouse Serum

Mouse CCL2/JE/MCP-1 capture antibody (#AF-479-NA), mouse CCL2/JE/MCP-1 recombinant protein (#479-JE), mouse CCL2/MCP-1 detection antibody (#BAF-479), mouse IL-6 capture antibody (#MAB-406), mouse IL-6 recombinant protein (#406-ML), and mouse IL-6 detection antibody (#BAF406) were used according to the manufacturer’s instructions (R&D Systems).

#### 2.2.3. Hematoxylin and Eosin Staining of Lung Tissue

H&E staining of mouse lung tissue was performed as described previously [26]. After lung tissues were isolated, they were fixed in 4% formaldehyde, embedded in paraffin wax, and sectioned into 5 μm thick slices. Lung tissues were then stained with H&E. Representative H&E images of lung tissue are shown.

##### Immunohistochemistry

IHC was performed as described previously [27]. Sections of lungs isolated from mice were deparaffinized, rehydrated, and washed three times with PBS. Sections were treated with primary antibodies (anti-F4/80 and SAB5500103, Sigma-Aldrich, St. Louis, MI, USA; anti-*S. aureus* antibodies, ab20920, Abcam, Cambridge, UK) overnight at 4 °C and washed with PBS. Sections were then treated with secondary antibodies for 30 min at 37 °C and washed 3 times for 5 min in PBS. After incubation with 0.05% (*w*/*v*) 3,3-diaminobenzidine tetrahydrochloride dehydrate, they were counterstained with hematoxylin and dehydrated. Dehydrated sections were mounted for examination using microscopy (Carl Zeiss Jena, Oberkochen, Germany), and images were captured using a Motic Digital Slide Assistant (Ver. 1.0.7.61b, Motic China Group Co., Ltd., Xiamen, China).

### 2.3. Cell Culture

A549 cells, adenocarcinomic human alveolar basal epithelial cell line, were purchased from Korean Cell Line Bank (KCLB 10185) and maintained in RPMI-1640 medium supplemented with 10% heat-inactivated fetal bovine serum (FBS) and 1% penicillin/streptomycin at 37 °C in a humidified 5% CO_2_ incubator. Cells were sub-cultured every 3 to 4 days. Cells were seeded onto 6- or 96-well plates and stabilized for 24 h before stimulation. Cells were then stimulated with the indicated dose of K8NPs and/or 10 ng/mL TNF-α and 10 ng/mL interferon gamma (IFN-γ) (denoted as TI) for the indicated times before the experiments were performed.

### 2.4. Cell Viability Assay

K8NPs were prepared at a concentration of 200 μg/mL (approximately 2 × 10^8^ CFU/mL) in Dulbecco’s PBS. A549 cells were stimulated with different concentrations of K8NPs and/or TI for 24 h. Cell viability was measured with an EZ-Cytox cell viability assay kit (Daeil Lab Service, Seoul, Republic of Korea) according to the manufacturer’s instructions. Absorbance was detected at a wavelength of 550 nm using a microplate reader (Eppendorf Biophotometer, Hamberg, Germany).

### 2.5. ELISA Analysis Using Cell Culture Supernatants 

A549 cells were stimulated with K8NPs and/or TI, and culture supernatants were collected and used in an enzyme-linked immunosorbent assay (ELISA) to detect CCL2 and IL-6. Human CCL2/MCP-1 capture antibody (#MAB206), human CCL2/MCP-1 recombinant protein (#279-ML), human CCL2/MCP-1 detection antibody (#BAF279), human IL-6 capture antibody (#MAB206), human IL-6 recombinant protein (#206-IL), and human IL-6 detection antibody (#BAF206) were used according to the manufacturer’s instruction (R&D Systems, Minneapolis, MN, USA). 

### 2.6. Western Blot Analysis

Cells were stimulated with a specified dose of K8NPs and/or TI for the indicated times, and cells were harvested using centrifugation for 10 min at 12,000× *g*. Pellets were lysed with a pro-prep cell lysis buffer (iNtRON Biotechnolog, Seongnam, Republic of Korea), and the protein concentration was quantified with a Bradford assay. Samples were mixed with a 2 × Laemmli buffer and boiled at 100 °C for 5 min. Sodium dodecyl sulfate-polyacrylamide gel electrophoresis was performed to separate proteins, which were transferred to a polyvinylidene fluoride membrane (Millipore, MA, USA). The membrane was blocked with 5% skim milk dissolved in Tris-buffered saline with 0.1% Tween 20 detergent (TBST) for 30 min at room temperature (RT). The membranes were incubated with the indicated primary antibodies, including anti-phospho-p38 (#9211S), anti-phospho-ERK (#9101S), anti-phospho-JNK (#9251S), and anti-phospho-NF-κB p65 (#3033S). These antibodies were purchased from Cell Signaling Technology (Danvers, MA, USA) and kept at 4 °C overnight, after which the membranes were washed three times with TBST. Secondary horseradish peroxide-conjugated anti-rabbit antibody (sc-2357, Santa Cruz Biotechnology, Santa Cruz, CA, USA) was incubated with membranes for 2 h at RT. After being washed three times with TBST, protein bands were produced with an ECL Pico Western blotting reagent (Thermo Fisher Scientific Inc., Waltham, MA, USA) and recorded on an X-ray film, with β-actin used as a loading control.

### 2.7. Statistical Analysis

All experiments were performed at least 3 times. The data shown are represented as means ± standard deviation. Statistical analyses were conducted with an unpaired two-tailed *t*-test and a one-way analysis of variance (ANOVA) followed by the Tukey’s honestly significant difference post hoc test, or a two-way ANOVA. Differences were considered significant at *p* < 0.05. GraphPad Prism 5 software (Ver. 5.01) was used for these analyses (GraphPad Software, Inc., San Diego, CA, USA).

## 3. Results

### 3.1. K8NPs Reduced S. aureus Lung Infection in Mice 

In a typical respiratory infection experiment, when a single dose of *S. aureus* is administered to healthy mice, the rate of mouse mortality depends on the inhaled *S. aureus* concentration. When 3.2 × 10^7^ CFU of *S. aureus* was inhaled through the nose, the mice did not die until the 7th day of the test. When more than 1 × 10^8^ CFU was inhaled, death occurred after the 4th day, and approximately 70% of the mice survived for more than 1 week. On the other hand, no adverse reactions such as death, gait disturbance, behavioral abnormalities, squatting, and diarrhea occurred when K8NPs were administered orally every day for 7 days at different doses of 1 × 10^6^, 1 × 10^7^, and 1 × 10^8^. When the oral administration of various concentrations of K8NPs and the nasal inhalation of 3.2 × 10^7^ CFU *S. aureus* were performed together, all populations survived. Therefore, we administered a low concentration (1 × 10^6^ CFU) and a high concentration (1 × 10^8^ CFU) of K8NPs for 4 weeks and then administered them nasally at 3 × 10^7^ CFU of *S. aureus* to investigate the infection and inflammation of the lungs. In general, when probiotics are taken for an extended period rather than in a single dose, they settle in the intestines and help maintain homeostasis. Therefore, we administered K8NPs for 4 weeks.

**Figure 1:**
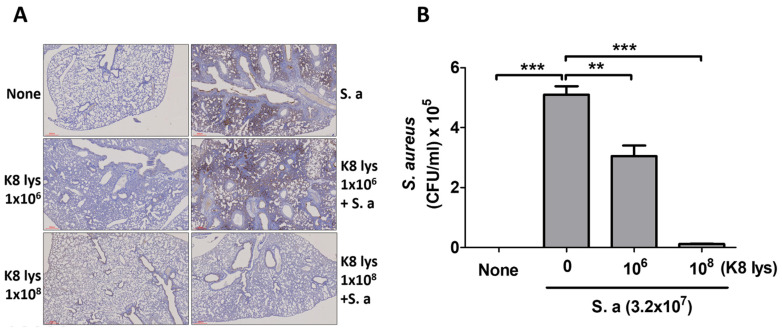
K8NPs inhibited *S. aureus* infection in the lung. Mice were orally administered an indicated dose of K8NPs (K8 lys) for 4 weeks and nasally infected with 3.2 × 10^7^ CFU of *S. aureus* for 1 day. After sacrificing the mice, lungs were isolated, and tissue samples were prepared for IHC analysis using anti-*S. aureus* antibody (ab20920, Abcam) (**A**) and CFUs were counted after the spreading of BALF onto a BHI agar plate (**B**). Statistical analysis was conducted with Student’s *t* test. ** *p* < 0.01, *** *p* < 0.001.

To examine whether K8NPs inhibit *S. aureus* lung infection, IHC analysis was performed with anti-*S. aureus* antibodies. *S. aureus* infection was seriously increased in the lungs of mice when nasally infected with *S. aureus* only, as well as when infected with *S. aureus* following the oral ingestion of low-concentration K8NPs for 4 weeks. However, the oral administration of high-concentration K8NPs effectively reduced *S. aureus* infection (Figure 1A). BALF was collected from the group administered K8NPs together with *S. aureus,* the *S. aureus*-only group, and the control group, and diluents were spread on BHI plates, and CFUs were counted after an overnight incubation. Compared with the *S. aureus*-only group, the number of CFUs from BALF was suppressed in both K8NP groups, although a low concentration of K8NPs did not reduce the *S. aureus* infection as much as the high-dose K8NPs (Figure 1B). Colonies growing on the plates were confirmed to be *S. aureus* using 16S rRNA sequencing. 

### 3.2. K8NPs Restored Alveolar Structure Damaged by S. aureus Infection

**Figure 2:**
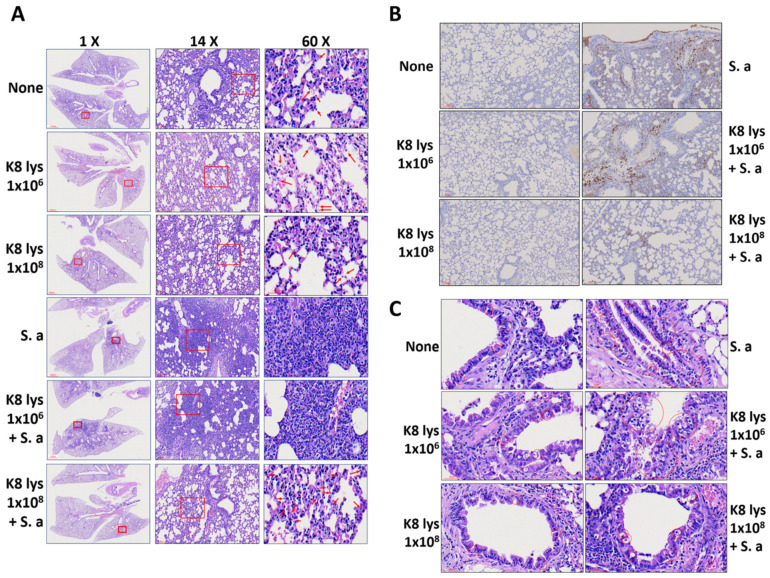
K8NPs recovered alveolar structure and inhibited the recruitment of macrophages in the lung. Mice were orally administered an indicated dose of K8NP (K8 lys), once a day for 4 weeks. The mice then inhaled *Staphylococcus aureus* (3.2 × 10^7^ CFU) intranasally and were cared for 1 day. After sacrificing the mice, the lungs were isolated, and tissue samples were prepared for H&E analysis (**A**), resident macrophage infiltration (**B**), and goblet cell analysis (**C**). The red box indicates the enlarged area, and the arrows indicate the immune cells. The red circles indicate goblet cells or the area of goblet cell group. Representative images are shown.

*S. aureus* infection caused structural changes in lung tissue. As shown in Figure 2A, 24 h after a nasal infection with *S. aureus*, the size and number of alveolar sacs and alveolar ducts decreased, making it difficult to distinguish the alveolar septum. This area was filled with lung cells and immune cells. There was a clear difference in the degree of recovery from *S. aureus*-induced lung damage when mice orally administered low concentrations of K8NPs (1 × 10^6^) and high concentrations of K8NPs (1 × 10^8^) for 4 weeks. The structure of the alveolus of the bronchiole of the mice fed a high concentration of K8NPs was restored to a near normal level, and the infiltration of immune cells was significantly suppressed compared with mice infected with *S. aureus* only (Figure 2A). After staining the mouse lung tissue with anti-F4/80 antibodies, the macrophage infiltration of lung tissue was measured. *S. aureus*-infected lung tissue showed a severe infiltration of macrophage cells into the alveolar sac and duct, an effect that was suppressed in mice fed a high concentration of K8NPs for 4 weeks. However, a low concentration of K8NPs did not have a significant effect on the inhibition of macrophage invasion (Figure 2B). In addition, lung infection with *S. aureus* induced an increase in the number of bronchiole goblet cells, which can accelerate the secretion of mucus, impair oxygen exchange, and can cause respiratory diseases such as asthma [28]. As shown in Figure 2C, the number of goblet cells increased in the pseudostratified epithelia of bronchioles after an *S. aureus* infection but decreased in *S. aureus*-infected mice fed a high concentration of K8NPs. In addition, the shape of the pseudostratified columnar ciliated epithelium was regularized in tissues in mice that ingested low or high concentrations of K8NPs only, while the shape of the pseudostratified columnar ciliated epithelium was broken in lung tissue infected with *S. aureus* (Figure 2C). These irregularities still appeared in tissues infected with *S. aureus* in mice fed a low concentration of K8NPs, whereas in mice fed a high concentration of K8NPs, the tissues appeared normal despite being infected with *S. aureus*, indicating that a high concentration of K8NPs restored alveolar structures damaged by an *S. aureus* infection.

### 3.3. Oral Administration of K8NPs Reduced CCL2 Production, Which Was Increased by S. aureus Infection or TI Injection 

**Figure 3:**
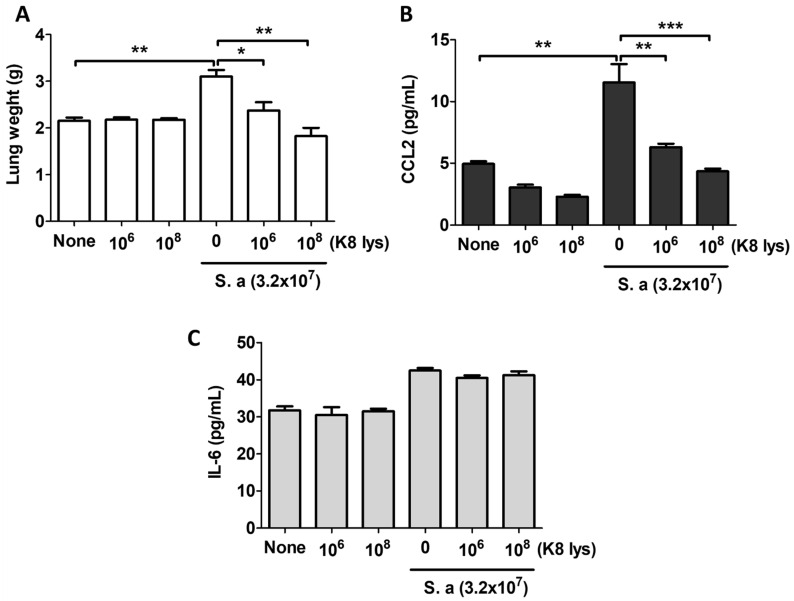
K8NPs inhibited *S. aureus*-induced CCL2 in mice. Mice were orally administered an indicated dose of K8NPs (K8 lys) for 4 weeks and nasally infected with 3.2 × 10^7^ CFU of *S. aureus* for 1 day. After sacrificing the mice, lungs were isolated, and the serum was collected for the analysis of cytokine expression. (**A**) The weight of an isolated lung was examined using a microbalance. The levels of CCL2 (**B**) and IL-6 (**C**) were examined using ELISA. Statistical analysis was conducted with Student’s *t* test. * *p* < 0.05, ** *p* < 0.01, *** *p* < 0.001.

*S. aureus* infection resulted in an increased lung weight. The lung weight of the *S. aureus*-infected mice was significantly increased compared with the untreated and K8NP only-treated groups, which decreased according to the intake concentration of K8NPs (Figure 3A). The increase in lung weight is thought to be due to the systemic inflammatory response caused by the *S. aureus* infection, which increased the expression of CCL2 in the blood. CCL2 is a representative inflammatory cytokine that activates monocytes and macrophages and recruits them into the inflammatory site (Figure 3B). The increased serum CCL2 in *S. aureus*-infected mice decreased in a dose-dependent manner according to the K8NPs ingested for 4 weeks. These results showed that K8NPs could inhibit the inflammatory response caused by *S. aureus*. IL-6 levels were slightly increased by *S. aureus*, but not to a significant degree. In addition, the intake of K8NPs did not affect the changes in blood IL-6 (Figure 3C).

### 3.4. K8NPs Inhibited TI-Induced CCL2 and IL-6 Production in A549 Cells

**Figure 4:**
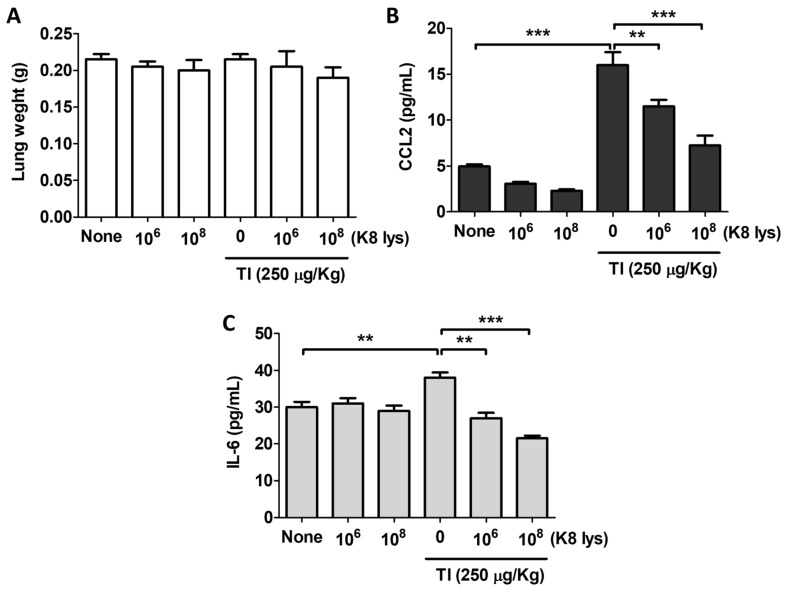
K8NPs inhibited TI-induced CCL2 and IL-6 in mice. Mice were orally administered an indicated dose of K8NPs (K8 lys) for 4 weeks and TI (250 μg/kg TNF-α and 250 μg/kg IFN-γ) was intraperitoneally injected, and the mice were cared for 1 day. After sacrificing the mice, lungs were isolated, and the serum was collected for analysis of cytokine expression. (**A**) The weight of an isolated lung was examined using a microbalance. The levels of CCL2 (**B**) and IL-6 (**C**) were examined using ELISA. Statistical analysis was conducted with Student’s *t* test. ** *p* < 0.01, *** *p* < 0.001.

To induce a systemic inflammatory response in mice, TI was injected intraperitoneally, and the changes in lung weight and blood cytokine levels were measured. Unlike the *S. aureus* infection, no change in lung weight was observed in either the TI-treated or TI + K8NP-treated group (Figure 4A). TI did not induce histological changes in mouse lungs, presumably because the exposure to TI was too brief to induce changes. However, TI injection significantly increased the expression of serum CCL2 and IL-6. CCL2 showed a more than three-fold increase in concentration after an injection of TI, and it decreased in a dose-dependent manner with the administration of K8NPs (Figure 4B). In the case of IL-6, the increase was small, but significant, and its serum level decreased depending on the administered concentration of pretreated K8NPs (Figure 4C). These results suggest that the *S. aureus* infection induced the production of inflammatory cytokines, and a chronic exposure to inflammatory cytokines can lead to respiratory diseases [8].

**Figure 5:**
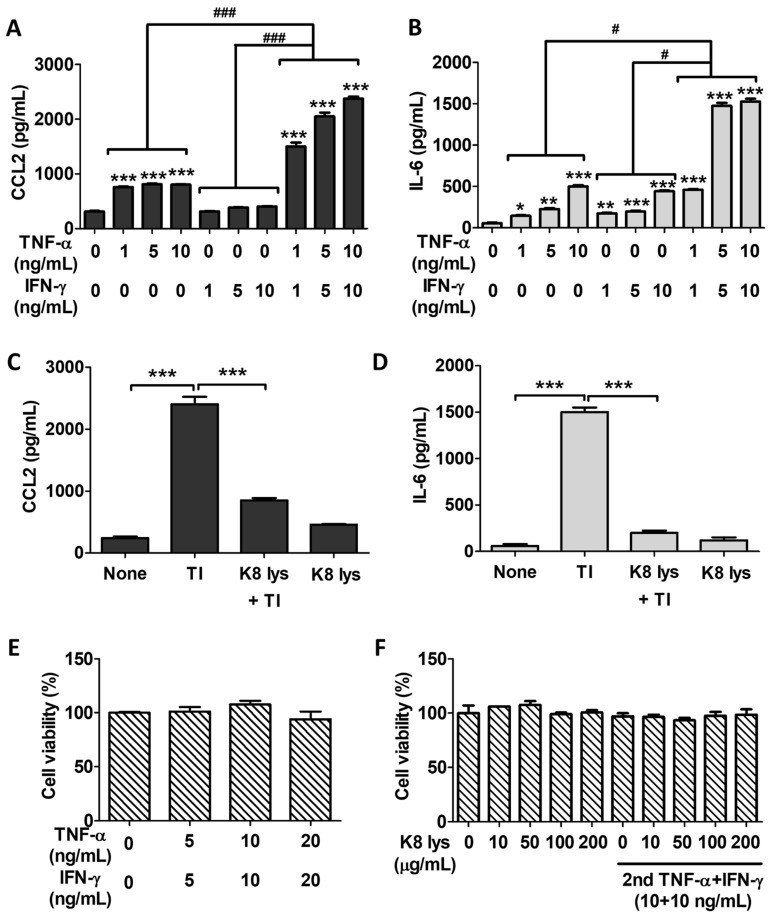
TI synergistically induced CCL2 and IL-6 in A549 cells. A549 cells were treated with indicated doses of TNF-α and/or IFN-γ for 24 h. The protein levels of CCL2 (**A**) and IL-6 (**B**) were examined using ELISA. Cells were pretreated with K8NPs for 18 h and then re-treated with TI (250 μg/kg TNF-α and 250 μg/kg IFN-γ) for 6 h. The protein levels of CCL2 (**C**) and IL-6 (**D**) were quantified using ELISA. Cell viability was examined with a WST-1 assay in A549 cells treated with TNF-α and IFN-γ (**E**) or a combination treatment of K8NPs and TI (**F**). Approximate concentrations shown (μg/mL) correspond to 10 = 3 × 10^6^, 50 = 1.5 × 10^7^, 100 = 3 × 10^7^, and 200 = 6 × 10^7^ CFU/mL. Statistical analysis was conducted with Student’s *t* test. * *p* < 0.05, ** *p* < 0.01, and *** *p* < 0.001 compared with untreated mice and two-way ANOVA. # *p* < 0.05, ### *p* < 0.001.

TI treatment synergistically induced the expressions of CCL2 and IL-6 compared with TNF-α or IFN-γ treatment alone. As shown in Figure 5, TI induced the expression of CCL2 in a dose-dependent manner in A549 cells, approximately 2.5 times higher than TNF-α alone and 6 times higher than IFN-γ alone at a concentration of 10 ng/mL (Figure 5A). Unlike the results of the in vivo study, the in vitro IL-6 expression was greatly increased by TI. Compared with TNF-α and IFN-γ alone, TI treatment showed an approximately 3-fold induction of IL-6 expression (Figure 5B). At the protein level, the secretions of CCL2 and IL-6 were increased by TI treatment, and the pretreatment with K8NPs significantly reduced the expression of these cytokines induced by TI (Figure 5C,D). TI treatment also induced cytotoxicity in some cells [29]. However, in A549 cells, no cytotoxicity was observed due to TI treatment or combination treatment with TI and K8NPs, suggesting that the inhibition of cytokine expression by K8NPs was not caused by cell death (Figure 5E,F).

### 3.5. Inhibition of Pro-Inflammatory Cytokines in TI-Treated A549 Cells Mediated by the Inactivation of Mitogen-Activated Protein Kinases and Nuclear Factor Kappa B 

**Figure 6:**
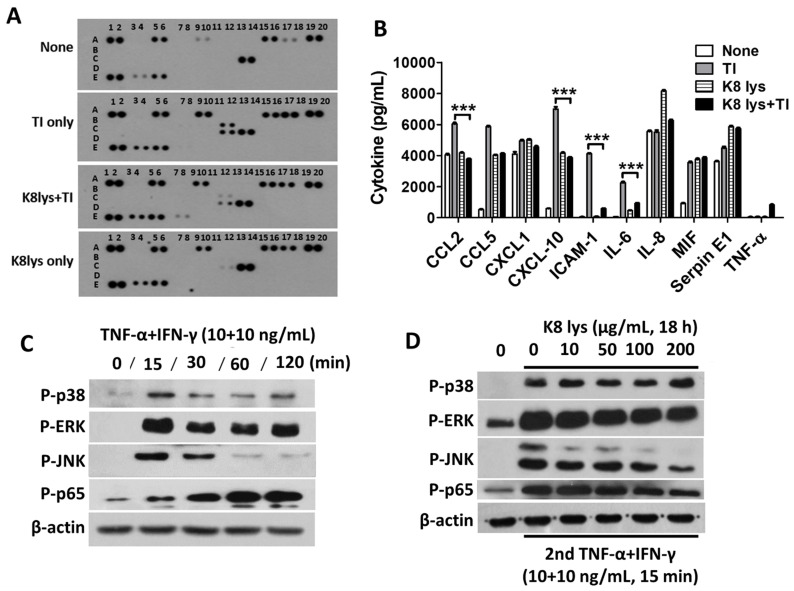
K8NPs inhibited TI-induced cytokine production through the reduction of NF-kB and MAPKs. (**A**,**B**) A549 cells were pretreated with K8NPs for 18 h and re-treated with TI (10 ng/mL TNF-α and 10 ng/mL IFN-γ) for 24 h. (**A**) Cytokine levels were examined with a Proteome Profiler Human Cytokine Array Kit (R&D Systems, Cat # ARY005B). (**B**) The expression level of cytokines was quantified using densitometry. Statistical analysis was conducted with Student’s *t* test. *** *p* < 0.001. (**C**) A549 cells were treated with TI for the indicated time, and the activation level of signaling molecules were examined with Western blots. (**D**) A549 cells were pretreated with an indicated dose of K8NPs for 18 h and then re-treated with TI for 15 min. The activation of signaling molecules were examined with Western blots. Approximate concentrations shown (μg/mL) correspond to 10 = 3 × 10^6^, 50 = 1.5 × 10^7^, 100 = 3 × 10^7^, and 200 = 6 × 10^7^ CFU/mL. In Figure 6A, A 1,2: reference spot; A 5,6: CCL2/MCP-1; A 9,10: CCL5/RATES; A 15,16: CXCL1/GRO-α; A 17,18: CXCL 10/IP-10; A 19,20: reference spot; B 11,12: ICAM-1/CD54; C 11,12: IL-6; C 13,14: IL-8; E 1,2: reference spot; E 3,4: MIF; E 5,6: Serpin E1/PAI-1; and E 7,8: TNF-α.

To observe the overall cytokine expression changes, a cytokine array using an A549 cell culture medium was performed after the treatment of TI and/or K8NPs (Figure 6A). Most of the cytokines used in the array were greatly increased by TI treatment, and among them, CCL2, CXCL-10, ICAM-1, and IL-6 were significantly decreased by K8NPs (Figure 6B). An increased cytokine expression due to TI was caused by the activation of mitogen-activated protein kinases (MAPKs) such as p38, ERK, JNK, and NF-kB. The activity of MAP-signaling substances was the strongest 15 min after TI treatment, after which it decreased gradually. The activity of the NF-kB p65 subunit increased gradually, reaching a maximum after 1 h treatment (Figure 6C). The inhibition of TI-induced cytokine expression by K8NP treatment occurred through the inhibition of these MAPKs and NF-kB activities (Figure 6D). As shown in Figure 6D, the activity of MAPKs decreased in a dose-dependent manner due to K8NPs at 15 min, and the NF-kB activity decreased in a dose-dependent manner due to K8NPs at 60 min.

## 4. Discussion

*S. aureus* is a pathogen associated with a wide range of infections affecting the respiratory tract, ranging from asymptomatic colonies to fulminant necrotizing pneumonia. During its colonization, *S. aureus* upregulates its surface proteins, such as protein A, that facilitate the colonization of host tissues by promoting its adhesion to the tissues. Membrane-damaging toxins include hemolysins, leukotoxin, and leucocidin that lyse eukaryotic cell membranes. Exotoxins, such as SAE-G, TSST-1, exfoliatin toxin, and Panton–Valentine leukocidin (PVL), damage host tissues and provoke disease symptoms [30]. On the other hand, *S. aureus* internalizes into host cells through its interaction with host cell surface receptors such as fibronectin and integrins [31]. After infection, *S. aureus* uses one of the regulatory systems, e.g., accessory gene regulator (Agr) system, that variably regulates the expression of surface proteins and secreted toxins including α-hemolysin, β-toxin, and Protein A. Agr plays the most important role in the intracellular persistence of *S. aureus* within various cell types [5]. *S. aureus* infection then significantly increases the activation of NF-κB, p38, ERK, and JNK, through Toll-like receptor 2 (TLR2), leading to the production of TNF-α, IL-1β, and IL-6 [32]. In the current study, K8NPs inhibited the activation of p38, ERK, and JNK in a dose-dependent manner, and the reductions in CCL2 and IL-6 were also observed. The literature and previous studies suggest that K8NPs inhibits the *S. aureus* infection and systemic inflammation through the modulation of host cells. 

In addition to interactions with host cells, the reduced infection of *S. aureus* in lung cells may be due to antibacterial peptides. K8NPs increase the expression of antibacterial peptides such as hBD2 and hBD3, and their expression leads to the inhibition of *S. aureus* infection in epithelial cells [33,34]. K8NPs stimulated the late signaling pathway, including JAK1/2, which may be induced by an EGFR- or IFN-γ–mediated pathway, and affected the induction of hBDs, which kills *S. aureus* and reduces its intracellular infection. In another study, we showed that K8NPs reduced the LPS-induced TNF-α production in THP-1 cells by down-regulating early MAPK and NF-κB signals [18]. One of the disadvantages of K8NPs is that it is not easy to select one substance that induces efficacy as it is a mixture of various substances. For that reason, the signal transduction mechanism is complicated, making it necessary to further subdivide the nanoparticles to study functional mechanisms. For example, lipoteichoic acid (LTA) and peptidoglycan (PGN) can be isolated and purified from bacterial lysates. As components of the cell wall of lactic acid bacteria, LTA and PGN play important roles in alleviating inflammatory diseases by inducing immune activities and suppressing excessive inflammatory responses, and related mechanisms have been studied in detail [35,36]. 

An excessive inflammatory response may induce respiratory diseases [8]. To test the induction of respiratory diseases by inflammatory reactions and the possibility of alleviation using K8NPs, TI was injected intraperitoneally 4 weeks after the oral administration of K8NPs, but no major abnormalities such as the alteration in alveolar cells, reduction in alveolar ducts, and increase in goblet cells were found upon a histological examination, indicating that a single injection of TI does not induce respiratory diseases in the lungs. However, the levels of CCL2 and IL-6 in the blood were significantly increased by TI and showed a tendency to decrease in a dose-dependent manner in mice that were administered K8NPs. These results suggest that TI can induce chronic inflammatory diseases by increasing the expression of CCL2 and IL-6, which are considered major inflammatory factors, and eventually cause long-term diseases such as pneumonia. They also show that taking K8NPs for an extended period can reduce the risk of chronic diseases by suppressing pro-inflammatory responses. TNF-α is one of the cytokines involved in inflammation and immune responses, and its expression is known to increase in inflammatory diseases. IFN-γ is an important cytokine in innate and adaptive immunities against viral, bacterial, and protozoal infections. While combined treatment with TNF-α and IFN-γ (TI) synergistically increases CCL2 and IL-6, this combination often causes a lethal cytokine shock in mice and cell death in bone marrow-derived macrophages (BMDMs) and THP-1 cells [37]. In the current study, we did not observe cell death caused by the TI combination, but it does affect cell signaling cascades and the gene expression associated with bacterial infection [38,39]. Infection with *S. aureus* creates a favorable environment for bacterial infection through an increased expression of host cell surface receptors. Simultaneously, TNF-α and IFN-γ, which are increased by *S. aureus*, not only have a synergistic effect on forming an infectious environment, but also induce a systemic inflammatory response, leading to organ dysfunction. Orally administered K8NPs can migrate to the intestines, induce changes in the intestinal microbiota, and regulate inflammation through interactions with intestinal epithelial cells and resident macrophages. In terms of the local inflammatory response, it is possible to control the pulmonary inflammatory response and control the expression of receptors and antibacterial peptides to suppress *S. aureus* infection in the lungs.

## 5. Conclusions

In conclusion, steroids are used to treat chronic respiratory diseases, but because a cure is yet to be found, research on functional materials that can replace steroids is being conducted. Long-term intake of K8NPs, a strain isolated from kimchi, can effectively suppress respiratory diseases caused by infection and chronic inflammation. In particular, K8NPs are expected to effectively prevent a progression to chronic diseases by alleviating asthma that may occur due to the *S. aureus* infection as well as by inhibiting the expression of excessive cytokines produced by inflammatory diseases. We expect that functional materials that help prevent or treat respiratory diseases can be developed using K8NPs.

## Data Availability

The data presented in this study are available on request from the corresponding authors.
